# Practice Patterns and Outcomes of Initial Anticoagulation Among Hospitalized Patients With Low- and Low-Intermediate-Risk Pulmonary Embolism

**DOI:** 10.1016/j.chpulm.2025.100151

**Published:** 2025-02-24

**Authors:** Grace M. Ferri, Om A. Kothari, Sarika D. Gurnani, Anica C. Law, Nicholas A. Bosch, Burton H. Shen

**Affiliations:** a Department of Medicine, Boston, MA; b Pulmonary Center, Boston University Chobanian and Avedisian School of Medicine and Boston Medical Center, Boston, MA

**Keywords:** anticoagulation, blood transfusion, heparin, pulmonary embolism

## Abstract

**Background:**

Guidelines recommend treatment with direct oral anticoagulants (DOACs) over unfractionated heparin (UFH) or low-molecular-weight heparin (LMWH) among ambulatory patients, including patients in the emergency department, with pulmonary embolism (PE) at low risk for mortality; however, recent evidence suggests that patients with low-risk PE are usually admitted to the hospital from the emergency department rather than discharged on DOACs.

**Research Question:**

Among hospitalized patients with low- and low-intermediate-risk PE, how do patterns in anticoagulation and outcomes vary between institutions?

**Study Design and Methods:**

This multicenter retrospective cohort study used the PINC AI enhanced administrative database (2016-2022). Eligible adult patients were admitted to a general ward, had an International Classification of Diseases, 10th Revision, diagnosis code for PE present on admission, were initiated on anticoagulation (UFH, LMWH/fondaparinux, or a DOAC) on but not before day 1, and had troponin and brain natriuretic peptide below the upper limit of normal. Initial anticoagulation practices were summarized overall and by hospital. Regression modeling was used to determine associations between initial anticoagulation and median length of stay.

**Results:**

Among 2,369 eligible patients, the percentage of patients initiated on UFH was 54%, initiated on LMWH/fondaparinux was 41%, and initiated on DOACs was 4%. Anticoagulation with DOACs decreased median length of stay by 0.62 days (95% CI, −1.04 to −0.20) compared with those who initially received UFH.

**Interpretation:**

Our results showed that hospitalized patients with low- and low-intermediate-risk PE generally do not receive initial DOACs. However, use of initial DOAC therapy was associated with shorter hospital length of stay compared with other initial anticoagulation strategies.

**Take-Home Points**

**Study Question:** Among inpatients with low- and low-intermediate-risk pulmonary embolism, how do patterns in treatment and outcomes vary between institutions?

**Results:** We found 54% of patients were initiated on unfractionated heparin, 41% on subcutaneous anticoagulation (low-molecular-weight heparin and fondaparinux), and 4% on a direct oral anticoagulant (DOAC); anticoagulation with DOACs decreased median length of stay by 0.62 days compared with a DOAC.

**Interpretation:** Hospitalized patients with low- and low-intermediate-risk pulmonary embolism usually receive unfractionated heparin or subcutaneous anticoagulation rather than DOACs, which prolong their length of stay.

More than 900,000 people in the United States are diagnosed with acute pulmonary embolism (PE) each year.^1^ Initial treatment of patients with incident PE is focused on anticoagulation to minimize clot propagation.^2^, ^3^, ^4^, ^5^, ^6^, ^7^ Guidelines recommend treatment with direct oral anticoagulants (DOACs) over unfractionated heparin (UFH) or low-molecular-weight heparin (LMWH) among ambulatory patients, including patients in the emergency department, with PE at low risk for mortality; however, evidence suggests that patients with low-risk PE are usually admitted to the hospital from the emergency department rather than discharged on DOACs and that all-comer hospitalized patients in 2020 with PE usually receive either UFH rather than DOACs.^2^^,^^7^, ^8^, ^9^, ^10^, ^11^ Thus, a large proportion of patients with low-risk PE who present to the emergency department are being admitted rather than discharged; once admitted, patients with PE typically initially receive UFH. However, it is unclear if high use of UFH in inpatient settings is warranted (among patients with higher risk PE, bleeding diatheses, planned interventional procedures) or potentially unwarranted (among patients with lower risk PE and no clear indication to receive UFH or LMWH instead of a DOAC). Thus, in this study, we sought to characterize patterns of anticoagulation and associated outcomes among hospitalized patients with hemodynamically stable, low- and low-intermediate-risk PE who had no clear indications for UFH or LMWH therapy.

## Study Design and Methods

### Study Population

We performed a multicenter retrospective cohort study using data from the PINC AI enhanced administrative database from 2016 to 2022.^12^ This source contains hospital-, patient-, and service-level data on 346 million patients from 1,054 different facilities.^12^ The aggregated, deidentified information on 9 million admissions per year included in the PINC AI database accounts for approximately 25% of annual US inpatient admissions.^12^ Hospital features include nonprofit, nongovernment, community, teaching, and safety-net institutions in different geographic regions across the United States.^12^ For this study, we used a subset of the data (10%) that includes patients from hospitals that contribute laboratory result data necessary for risk-stratifying PE.

Eligible patients with low- or low-intermediate-risk PE were defined as adults (aged 18-79 years) who were admitted to the general ward on hospital day 1, had a primary International Classification of Diseases, 10th Revision, diagnosis code for other pulmonary embolism without acute cor pulmonale (I26.99) or subsegmental pulmonary embolism [or emboli] without acute cor pulmonale (I26.93 and I26.94) present on admission, and had troponin and brain natriuretic peptide values below the upper limit of normal (e-Appendix1). Patients were excluded if they had diagnoses or procedures on admission that could (1) make patients more likely to receive initial UFH or LMWH or (2) suggest previous use of anticoagulation therapy: chronic thromboembolism, heparin-induced thrombocytopenia, pregnancy, intracranial infarct or hemorrhage, gastrointestinal hemorrhage, liver failure, surgical attending physician, invasive procedures (lumbar puncture, paracentesis, abdominal wall drainage, thoracentesis, and central line insertion) or major surgery, thrombectomy, use of lytic medication, use of vasopressors, or transfer from the wards to the intermediate care unit or ICU on the day of hospital admission (e-Appendix 1). Patients were also excluded if they had a glomerular filtration rate ≤ 30 mL/min on hospital day 1 because these patients may have preferentially received initial UFH. We subsequently limited the cohort to patients who received initial anticoagulation on hospital day 1, which was defined using charge codes for UFH, subcutaneous anticoagulation (LMWH or fondaparinux), or a DOAC.

### Initial Anticoagulation Use

From each patient, we classified initial anticoagulation into categories of UFH, subcutaneous anticoagulation, or DOAC, where patients who received UFH were considered to be in the reference cohort. For patients who received > 1 anticoagulant on hospital day 1, patients were assigned an initial anticoagulation regimen as follows: if received UFH and any other anticoagulant, assigned to UFH; if received a subcutaneous anticoagulation and a DOAC, assigned to subcutaneous anticoagulation. We chose to assign initial anticoagulation regimens this way under the assumption that patients would be unlikely to transition from a DOAC to a subcutaneous regimen or from subcutaneous anticoagulation to UFH. We also obtained the duration of initial anticoagulation regimen, anticoagulation regimen on the day of hospital discharge, and time from initial anticoagulation regimen initiation to oral treatment initiation.

### Outcomes

The primary outcome was hospital length of stay in days, starting from hospital day 1 until hospital discharge. We selected hospital length of stay as the primary outcome because this measure captures both delays in discharge secondary to slow transitions from a subcutaneous or IV anticoagulation to an outpatient appropriate oral regimen and delays in discharge secondary to a less effective anticoagulation. Secondary outcomes included escalation of care (defined as any transfer to an intermediate care unit or ICU after hospital day 1 until hospital discharge), blood transfusion use (a marker of bleeding; defined as any blood transfusion after hospital day 1 until hospital discharge), and hospital mortality (defined as death or discharge to hospice on any hospital day).

### Baseline Covariables

We accounted for variables that could confound the relationship between initial anticoagulation selection and outcomes. Included patient-level covariables were age, sex, race, discharge year, COVID-19 present on admission, metastatic cancer present on admission, cardiac arrhythmias present on admission, Gagne index for mortality, use of noninvasive mechanical ventilation on hospital day 1, use of invasive mechanical ventilation on hospital day 1, blood transfusion on hospital day 1, warfarin use on hospital day 1, and number of anticoagulants used on hospital day 1. Included hospital-level covariables were US Census region, safety net status, hospital bed size, whether the hospital was classified as urban or rural, and whether the hospital was a teaching hospital. Sensitivity analyses were performed among those with vital sign data available that would allow them to more likely meet criteria for low-risk PE (maximum heart rate < 110 beats/min and minimum oxygen saturation ≥ 90%) on day 1 of hospitalization.

### Statistical Analysis

We summarized baseline covariables using counts (proportions) for categorical data and means ± SDs or medians (interquartile ranges [IQRs]) for continuous data as appropriate. We calculated the proportion of patients who were started on each initial anticoagulation regimen on hospital day 1 overall and by hospital. We reported the median duration (in days) of initial anticoagulation regimen, the proportion of patients receiving each anticoagulation regimen on the day of hospital discharge, and the time from initial anticoagulation regimen initiation to oral treatment initiation (in days) by initial treatment regimen.

For the primary outcome, we reported the unadjusted median (IQR) hospital length of stay for each anticoagulation regimen and then used a quantile regression model that included anticoagulation regimen and all covariables to determine the association between initial anticoagulation regimen and hospital length of stay. We planned to use hierarchical quantile regression to account for hospital clustering but were unable to due to lack of model convergence. Effect estimates describing the association between initial anticoagulation regimen and hospital length of stay were reported as the adjusted median difference in length of stay between patients initially started on each anticoagulation regimen vs the reference regimen of initial UFH. For secondary outcomes, we first reported unadjusted proportions by initial anticoagulation regimen and then used hierarchical logistic regression models including initial anticoagulation regimen and all covariables as fixed effects and a random effect (random intercept) hospital identifier variable to account for clustering. Effect estimates from the hierarchical logistic regression models were reported as adjusted ORs (aORs) between patients initially started on each anticoagulation regimen vs the reference regimen of initial UFH. A low outcome event rate prevented model convergence and thus precluded calculation of aORs for the outcome of hospital mortality. All analyses were performed in R (version 4.0.5). Alpha was set at .05 for all comparisons. This study was designated not human patients research by the Boston University institutional review board (No. H-41795). This study followed the Strengthening the Reporting of Observational Studies in Epidemiology reporting guidelines.^13^ Analysis was performed on data from January 2016 to September 2022.

## Results

### Study Population

**Figure 1:**
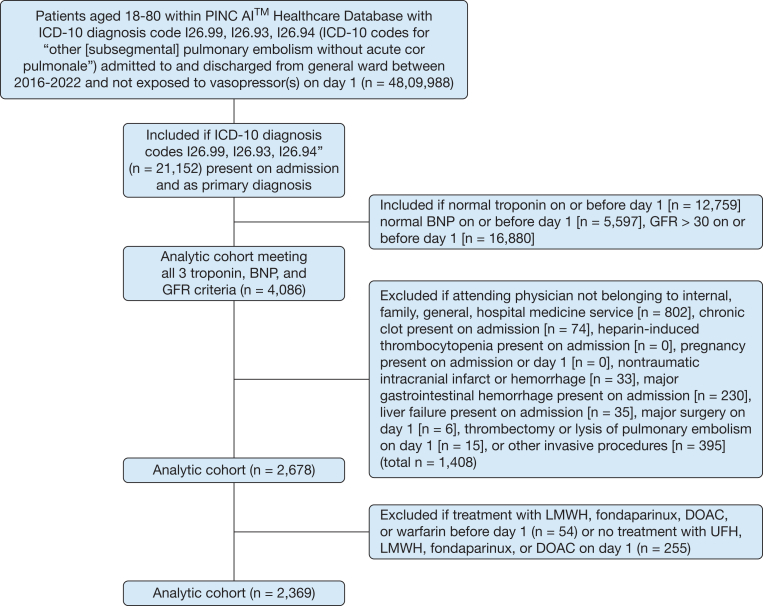
Consolidated Standards of Reporting Trials diagram. BNP = brain natriuretic peptide; DOAC = direct oral anticoagulant; GFR = glomerular filtration rate; ICD-10 = International Classification of Diseases, 10th Revision; LMWH = low-molecular-weight heparin; UFH = unfractionated heparin.

From January 2016 to September 2022, 2,369 patients with low- or low-intermediate-risk PE from 180 hospitals met inclusion criteria (Fig 1). The median age was 60 years (IQR, 49-70) (Table 1). One-half of patients (48%) were male, and most patients self-identified as being of White race (73%). Most patients were admitted to hospitals located in the southern (60%) or northeastern (24%) United States. Approximately 20% of patients were at institutions designated as safety-net hospitals.

**Table 1:** Baseline Patient and Hospital Characteristics

Characteristic	Overall (N = 2,369)	DOAC (n = 105)	Subcutaneous Anticoagulation (n = 978)	UFH (n = 1,286)
Age, y	60 (49-70)	60 (50-69)	60 (49-70)	61 (49-70)
Male sex	1,142 (48.2)	49 (46.7)	464 (47.4)	629 (48.9)
Race				
Asian	26 (1.1)	0 (0.0)	12 (1.2)	14 (1.1)
Black	480 (20.3)	16 (15.2)	209 (21.4)	255 (19.8)
Other	96 (4.1)	8 (7.6)	31 (3.2)	57 (4.4)
Unknown	33 (1.4)	1 (1.0)	15 (1.5)	17 (1.3)
White	1,734 (73.2)	80 (76.2)	711 (72.7)	943 (73.3)
Year				
2016	173 (7.3)	5 (4.8)	80 (8.2)	88 (6.8)
2017	232 (9.8)	12 (11.4)	115 (11.8)	105 (8.2)
2018	316 (13.3)	16 (15.2)	145 (14.8)	155 (12.1)
2019	377 (15.9)	13 (12.4)	166 (17.0)	198 (15.4)
2020	481 (20.3)	20 (19.0)	195 (19.9)	266 (20.7)
2021	501 (21.1)	23 (21.9)	179 (18.3)	299 (23.3)
2022	289 (12.2)	16 (15.2)	98 (10.0)	175 (13.6)
Region				
Midwest	338 (14.3)	14 (13.3)	69 (7.1)	255 (19.8)
Northeast	575 (24.3)	35 (33.3)	205 (21.0)	335 (26.0)
South	1,429 (60.3)	53 (50.5)	692 (70.8)	684 (53.2)
West	27 (1.1)	3 (2.9)	12 (1.2)	12 (0.9)
Teaching hospital	1,136 (48.0)	48 (45.7)	430 (44.0)	658 (51.2)
Safety-net hospital	478 (20.2)	27 (25.7)	148 (15.1)	303 (23.6)
Beds				
0-99	261 (11.0)	12 (11.4)	131 (13.4)	118 (9.2)
100-199	322 (13.6)	9 (8.6)	162 (16.6)	151 (11.7)
200-299	301 (12.7)	23 (21.9)	96 (9.8)	182 (14.2)
300-399	384 (16.2)	19 (18.1)	177 (18.1)	188 (14.6)
400-499	239 (10.1)	6 (5.7)	77 (7.9)	156 (12.1)
≥ 500	862 (36.4)	36 (34.3)	335 (34.3)	491 (38.2)
Urban	2,002 (84.5)	87 (82.9)	824 (84.3)	1,091 (84.8)
COVID-19-positive on admission	35 (1.5)	0 (0.0)	19 (1.9)	16 (1.2)
Metastatic cancer on admission	179 (7.6)	8 (7.6)	67 (6.9)	104 (8.1)
Cardiac arrhythmia	320 (13.5)	22 (21.0)	126 (12.9)	172 (13.4)
Gagne index combined on admission	2 (1-3)	2 (1-4)	2 (1-3)	2 (1-3)
Noninvasive ventilation on day 1	47 (2.0)	3 (2.9)	20 (2.0)	24 (1.9)
Invasive mechanical ventilation on day 1	6 (0.3)	0 (0.0)	3 (0.3)	3 (0.2)
Transfusion on day 1	5 (0.2)	1 (1.0)	1 (0.1)	3 (0.2)
Warfarin on day 1	98 (4.1)	0 (0.0)	56 (5.7)	42 (3.3)
2 Treatments on day 1	205 (8.7)	0 (0.0)	65 (6.6)	140 (10.9)

Values are No. (%) or median (interquartile range). DOAC = direct oral anticoagulant; Subcutaneous anticoagulation = low-molecular-weight heparin/fondaparinux; UFH = unfractionated heparin.

### Anticoagulation Regimen Practices

**Figure 2:**
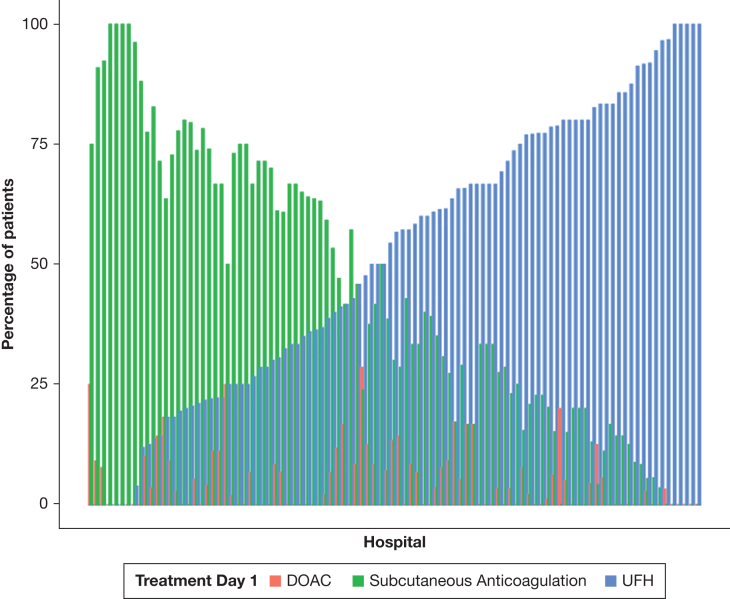
Anticoagulation modality on day 1 of treatment by hospital. Shown are the percentage of patients at each hospital that use each anticoagulant as initial therapy on hospital day 1. DOAC = direct oral anticoagulant; Subcutaneous anticoagulation = low-molecular-weight heparin/fondaparinux; UFH = unfractionated heparin.

Among the 2,369 included patients, 2,164 (91%) received 1 anticoagulant and 205 (9%) received 2 different anticoagulants on day 1. Of the patients who received 1 treatment on day 1, 53% received UFH, 42% received subcutaneous anticoagulation, and 5% received a DOAC. After reclassifying patients who received 2 anticoagulants on hospital day 1, a total of 1,286 (54%) received initial UFH, 978 (41%) received initial subcutaneous anticoagulation, and 105 (4%) received initial DOAC. Binning by hospital (n = 99), the median hospital-level use of DOACs on day 1 was 3% (IQR, 0%-8%). There were no hospitals in which DOACs were used as an initial anticoagulant more frequently than other anticoagulants (Fig 2).

In a sensitivity analysis including only those meeting criteria for low-risk (rather than low-intermediate-risk) PE (maximum heart rate < 110 beats/min and minimum oxygen saturation ≥ 90) on day 1 of hospitalization (n = 234), 65% received UFH (n = 151), 32% received subcutaneous anticoagulation (n = 74), and 4% received a DOAC (n = 9).

On the day of discharge, most patients received a DOAC (60%) (e-Fig 1). DOAC use on discharge depended on the initial treatment received: 88% of patients initially receiving DOAC were discharged on DOAC vs 64% of patients initially receiving UFH and 50% of patients initially receiving subcutaneous anticoagulation. The median duration of initial treatment for all patients was 2 days (IQR, 2-3). Patients who were initially treated with UFH or subcutaneous anticoagulation had shorter durations of their initial therapy (median, 2 days [IQR, 1-3] and 2 days [IQR, 2-3], respectively) than those initially treated with DOACs (median, 3 days [IQR, 2-4]).

### Patient Outcomes

The median hospital length of stay was 3 (IQR, 2-4), 2 (IQR, 2-3), and 2 (IQR, 2-3) days in patients who received initial UFH, subcutaneous anticoagulation, and DOACs, respectively (Table 2). In the adjusted models, patients receiving DOACs had a median length of stay 0.62 days shorter (95% CI, −1.04 to −0.20) than those who initially received UFH (Table 3). Patients who initially received subcutaneous anticoagulation also had shorter lengths of stay than those who received UFH (−0.44 days; 95% CI, −0.71 to −0.17). In a sensitivity analysis including only those meeting criteria for low-risk (rather than low-intermediate-risk) PE on day 1 of hospitalization (n = 234), median hospital length of stay was 2 (IQR, 2-3), 2 (IQR, 1-3), and 2 (IQR, 1-2) days in patients who received initial UFH, subcutaneous anticoagulation, and DOACs, respectively.

**Table 2:** Unadjusted Outcomes by Individual-Level Treatments

Outcome	Treatment on Hospital Day 1			
	Any Treatment	UFH	Subcutaneous Anticoagulation	DOAC
LOS, d	2 (2-4)	3 (2-4)	2 (2-3)	2 (2-3)
Duration of day 1 treatment, d	2 (2-3)	2 (1-3)	2 (2-3)	3 (2-4)
Time to oral treatment, d (n = 1,713)^a^	2 (2-3)	2 (2-3)	2 (2-3)	1 (1-1)
Treatment on day of discharge^a^^,^^b^	2,357 (99.5)	1,279 (99.5)	973 (99.5)	105 (100.0)
UFH	437 (18.4)	416 (32.3)	19 (1.9)	2 (1.9)
Subcutaneous anticoagulation	755 (31.9)	238 (18.5)	507 (51.8)	10 (9.5)
DOAC	1,405 (59.3)	821 (62.8)	492 (50.3)	92 (87.6)
Warfarin	123 (5.2)	74 (5.8)	45 (4.6)	4 (3.8)
Blood transfusion	37 (1.6)	15 (1.2)	17 (1.7)	5 (4.8)
Days of blood transfusion	0 (0-0)	0 (0-0)	0 (0-0)	0 (0-0)
Hospital mortality	7 (0.3)	6 (0.5)	1 (0.1)	0 (0.0)
Upgrade in care^c^	129 (5.4)	82 (6.4)	43 (4.4)	4 (3.8)

Values are presented as No. (%) or median (interquartile range). DOAC = direct oral anticoagulant; LOS = length of stay; Subcutaneous anticoagulation = low-molecular-weight heparin/fondaparinux; UFH = unfractionated heparin.

a Warfarin or DOAC (among those treated with oral anticoagulation).

b Categories not mutually exclusive.

c ICU or stepdown bed.

**Table 3:** Adjusted Outcomes by Individual-Level Treatments

Outcome	UFH	Subcutaneous Anticoagulation	DOAC
LOS, median difference (95% CI), d	Ref	−0.44 (−0.71 to −0.17)	−0.62 (−1.04 to −0.20)
Duration of initial treatment – median difference (95% CI), d	Ref	0.20 (0.05 to 0.35)^a^	0.70 (0.31 to 1.08)^a^
Time to oral treatment (n = 1,713), median difference (95% CI), d	Ref	−0.34 (−0.52 to −0.16)^a^	−1.74 (−1.91 to −1.58)^a^
Blood transfusion, aOR (95% CI)	Ref	1.82 (0.85 to 3.89)	4.04 (1.30 to 12.54)
Upgrade in care, aOR (95% CI)	Ref	0.57 (0.34 to 0.95)	0.38 (0.10 to 1.40)

aOR = adjusted OR; DOAC = direct oral anticoagulant; LOS = length of stay; Ref = reference; Subcutaneous anticoagulation = low-molecular-weight heparin/fondaparinux; UFH = unfractionated heparin.

a Removed all covariates except age and Gagne index score because of convergence problems.

For secondary outcomes, the rate of blood transfusion was highest among those receiving DOACs (4.8%) compared with those receiving UFH (1.2%) or subcutaneous anticoagulation (1.7%). After adjusting for patient and hospital variables, patients receiving DOACs had > 4 times higher odds of receiving blood transfusions during hospitalization than those on UFH (aOR, 4.04; 95% CI, 1.30-12.54). Rates of blood transfusion among those on subcutaneous anticoagulation compared with UFH (aOR, 1.82; 95% CI, 0.85-3.89) were imprecise. A total of 6 patients (0.5%) treated with UFH died in the hospital vs 0 (0.0%) of those treated with a DOAC and 1 (0.1%) of those treated with subcutaneous anticoagulation; 82 patients (6.4%) receiving UFH on day 1, 43 patients (4.4%) receiving subcutaneous anticoagulation on day 1, and 4 patients (3.8%) receiving DOACs on day 1 required a higher level of care during hospitalization (Table 2). In the adjusted analyses, only subcutaneous anticoagulation use—not DOACs—resulted in a statistically significant increased odds of upgrade in care (aOR, 0.57; 95% CI, 0.34-0.95) (Table 3).

## Discussion

Although guidelines surrounding the use of DOACs for PE are predominantly applied to patients in the ambulatory setting, we sought to study the patients admitted to the hospital with PE not requiring intensive care hospitalization or proceduralization. We used a large multicenter cohort of US inpatient admissions to quantify the initial anticoagulation practices among these patients with low- and low-intermediate-risk PE. Most patients in our study initially received UFH or subcutaneous anticoagulation, despite usually receiving DOACs on discharge. Use of initial DOACs was interestingly associated with shorter length of stay (0.62 days) than UFH. This observation may be particularly notable when considering the median overall length of stay was 2 days, suggesting a 45% relative effect.

This study should be considered in the context of prior work. Unlike previous studies analyzing anticoagulation trends among all-comer patients admitted with PE, our work is the first, to our knowledge, to stratify patients by low- and low-intermediate-risk status (using presence of normal vital signs, troponin, and brain natriuretic peptide levels) and assess subsequent anticoagulation choices in an era of novel therapeutics (after 2016).^8^ Our analysis provides stronger evidence that, among patients with the clearest indication for a DOAC, clinicians continue to primarily administer initial UFH or subcutaneous anticoagulation in the inpatient setting.^14^, ^15^, ^16^ Future research should aim to understand whether ambulatory guidelines for anticoagulation in low-risk PE can be extrapolated to the inpatient setting. Given higher in-hospital transfusion use (possibly suggesting higher bleeding rates) observed among patients receiving initial DOAC, future studies should also seek to assess if the risk-to-benefit tradeoff of preliminary DOAC use is exclusive to hospitalized patients or similarly present among outpatients.

This study has limitations. First, administrative databases are prone to misclassification. To mitigate this possibility, we used not only International Classification of Diseases, 10th Revision, diagnosis codes but also laboratory values (brain natriuretic peptide, troponin, glomerular filtration rate) and inpatient unit and service (general ward, nonsurgical attending) to most appropriately select a cohort of those with low- or low-intermediate risk PE. Despite our exclusion criteria and model adjustment, we must acknowledge the potential for unmeasured confounding, where patients considered to be severely ill by other metrics likely would have been deemed inappropriate candidates for DOAC therapy. Second, our study only looked at admitted patients. Unfortunately, we did not have access to prehospitalization data including time spent in the emergency department setting. Although guidelines recommend that patients with low-risk PE be treated as outpatients, two-thirds of patients with low-risk PE continue to be admitted.^9^ As a result, inpatients with low-risk PE lack uniform management recommendations.^7^^,^^14^^,^^15^^,^^17^, ^18^, ^19^, ^20^ Third, in terms of the hospitals surveyed, most patients included in this study were at urban institutions in the southern or northeastern United States, thereby limiting the generalizability of our conclusions to patients admitted to hospitals in rural areas or other parts of the United States. However, the hospitals we surveyed were heterogeneous in size.^21^ Moreover, the PINC AI database features nearly 25% of US inpatient admissions per year from across the country and thus provides a cross section of US admissions.^12^

## Interpretation

Most patients who were hospitalized for low-risk PE were not initially started on a DOAC. Those who were initiated on a DOAC had a shorter overall length of stay than those on UFH, with no significant differences in in-hospital mortality or requiring an increased level of care compared with those on UFH or subcutaneous anticoagulation.

## Funding/Support

This work was supported by the ASH HONORS Award for Residents (to G. M. F.), the 10.13039/100000968American Heart Association [Grant 24CDA1267699] (to N. A. B.), and the National Heart, Lung, and Blood Institute T32HL007035 (to B. H. S.).

## Financial/Nonfinancial Disclosures

None declared.

## Figures and Tables

**e-Online Data:**
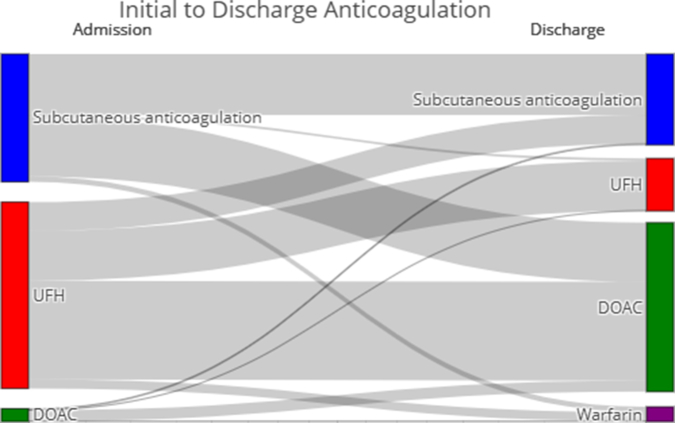


## Supplementary Data

e-Online Data

e-Online Data
