# Supplementary material for: Practice Patterns and Outcomes of Initial Anticoagulation Among Hospitalized Patients With Low- and Low-Intermediate-Risk Pulmonary Embolism
Source: CHEST Pulm. 2025 Feb 24;3(2):100151. doi: 10.1016/j.chpulm.2025.100151 (PMC12330347; doi:10.1016/j.chpulm.2025.100151)
Supplement: e-Online Data [file mmc1.docx]

**Practice Patterns and Outcomes of Initial Anticoagulation Among Hospitalized Patients With Low-Risk Pulmonary Embolism**

**Authors:**

Grace M. Ferri^1*^, Om A. Kothari^1*^, Sarika D. Gurnani^1^, Anica C. Law^2^, Nicholas A. Bosch^2^, Burton H. Shen^2^

**Supplemental Material:**

**e-Appendix 1:**

*Other pulmonary embolism without acute cor pulmonale ICD-10 code:* "I26.99"

*Single subsegmental thrombotic pulmonary embolism without acute cor pulmonale ICD-10 code:* "I26.93"

*Multiple subsegmental thrombotic pulmonary emboli without acute cor pulmonale ICD-10 code:* "I26.94"

*Chronic embolism ICD-10 codes:* "I82.211", "I82.221", "I82.291", "I82.501" , "I82.502", "I82.503", "I82.509", "I82.511", "I82.512", "I82.513", "I82.519", "I82.521", "I82.522", "I82.523", "I82.529", "I82.531", "I82.532", "I82.533", "I82.539", "I82.541", "I82.542", "I82.543", "I82.549", "I82.551", "I82.552", "I82.553", "I82.559", "I82.561", "I82.562", "I82.563", "I82.569", "I82.591", "I82.592", "I82.593", "I82.599", "I82.5Y1", "I82.5Y2", "I82.5Y3", "I82.5Y9", "I82.5Z1", "I82.5Z2", "I82.5Z3", "I82.5Z9", "I82.701", "I82.702", "I82.703", "I82.709", "I82.711", "I82.712", "I82.713", "I82.719", "I82.721", "I82.722", "I82.723", "I82.729", "I82.A21", "I82.A22", "I82.A23", "I82.A29", "I82.B21", "I82.B22", "I82.B23", "I82.B29", "I82.C21", "I82.C22", "I82.C23", "I82.C29", "I82.891", "I82.91", "I27.82"

*Heparin-induced thrombocytopenia ICD-10 codes:* "D75.821", "D75.822", "D75.828", "D75.829"

*Pregnancy ICD-10 codes:* "Z38.8", "Z38.7", "Z38.4", "Z38.5", "Z38.2", "Z38.1", "Z37.2", "Z37.0", "Z37.3", "O77.9", "O77.8", "O70.9", "O70.3", "O70.1", "O70.0", "O77.0", "O75.9", "O75.5", "O74.9", "O74.8", "O74.7", "O74.6", "O74.5", "O74.4", "O74.3", "O74.2", "O74.1", "O74.0", "O70.4", "Z37.9", "O75.1", "O75.0", "O63.2", "O67.9", "O67.8", "O67.0", "O25.2", "O16.4", "O13.4", "O11.4", "O66.5", "O77.1", "O04.6", "O04.7", "Z33.2", "Z33.2", "O04.5", "Z36.3", "O48.0", "O48.1", “O82.", "O80.", "O76.", "O68.", "O02.1", "O02.0", "O02.9", "O03.6", "O03.1", "O03.7", "O03.2", "O03.9", "O03.4", "Z38.69", "Z38.68", "Z38.66", "Z38.65", "Z38.64", "Z38.63", "Z38.62", "Z38.61", "Z38.31", "Z38.30", "Z38.01", "Z38.00", "Z37.59", "Z37.54", "Z37.53", "Z37.52", "Z37.51", "Z37.50", "O75.82", "Z37.69", "Z37.64", "Z37.63", "Z37.62", "Z37.61", "Z37.60", "O99.12", "O70.23", "O70.22", "O70.21", "O70.20", "O75.89", "O75.81", "O9A.52", "O9A.42", "O9A.32", "O9A.22", "O9A.12", "O99.72", "O99.62", "O99.52", "O99.42", "O98.92", "O98.82", "O98.72", "O98.62", "O98.52", "O98.42", "O98.22", "O98.12", "O98.02", "O88.82", "O88.32", "O88.22", "O88.12", "O88.02", "O26.62", "O26.72", "O24.92", "O24.82", "O24.32", "O24.12", "O24.02", "O14.94", "O14.24", "O14.14", "O14.04", "O12.24", "O12.14", "O12.04", "O10.92", "O10.42", "O10.32", "O10.22", "O10.12", "O10.02", "O42.92", "O42.12", "O42.02", "O04.84", "O04.82", "O04.83", "O04.81", "O04.85", "O04.86", "O04.88", "O04.89", "O04.80", "O02.81", "O02.89", "O03.84", "O03.34", "O03.82", "O03.32", "O03.83", "O03.33", "O03.81", "O03.31", "O03.85", "O03.86", "O03.88", "O03.89", "O03.35", "O03.36", "O03.38", "O03.39", "O03.80", "O03.30", "Z34.01", "Z34.81", "Z34.91", "Z32.01", "O09.01", "O09.11", "O09.A1", "O09.41", "O09.31", "O09.71", "O09.91", "O09.41", "Z34.02", "Z34.82", "Z34.92", "O09.42", "O09.02", "O09.12", "O09.A2", "O09.42", "O09.32", "O09.72", "O09.92", "Z34.03", "Z34.83", "Z34.93", "O09.43", "O09.03", "O09.13", "O09.A3", "O09.43", "O09.33", "O09.73", "O09.93", "Z34.00", "Z34.90", "Z34.80", "O09.70", "O09.90", "O09.40", "O09.10", "O09.00", "O09.A0", "O09.30", "P07.21", "P07.01", "P07.00", "P07.02", "P07.03", "P07.14", "P07.15", "P07.16", "P07.17", "P07.18", "P07.30", "P07.10", "P07.20", "P08.21", "P08.22", "Z3A.49", "P07.22", "P07.23", "P07.24", "P07.25", "P07.26", "P07.31", "Z3A.20", "Z3A.21", "Z3A.22", "Z3A.23", "Z3A.24", "Z3A.25", "Z3A.26", "Z3A.27", "Z3A.28", "Z3A.29", "P07.32", "Z3A.30", "P07.33", "Z3A.31", "P07.34", "Z3A.32", "P07.35", "Z3A.33", "P07.36", "Z3A.34", "P07.37", "Z3A.35", "P07.38", "Z3A.36", "P07.39", "Z3A.37", "Z3A.38", "Z3A.39", "Z3A.40", "Z3A.41", "Z3A.42", “O99.844", "O99.354", "O99.334", "O99.214", "O99.834", "O99.824", "O99.814", "O99.344", "O99.324", "O99.314", "O99.284", "O24.429", "O24.425", "O24.424", "O24.420", "O09.291", "O09.211", "O09.291", "O09.511", "O09.521", "O09.611", "O09.621", "O09.811", "O09.821", "O09.891", "O09.511", "O09.521", "O09.512", "O09.522", "O09.212", "O09.292", "O09.512", "O09.522", "O09.612", "O09.622", "O09.812", "O09.822", "O09.892", "O09.513", "O09.523", "O09.213", "O09.293", "O09.513", "O09.523", "O09.613", "O09.623", "O09.813", "O09.823", "O09.893", "O09.529", "O09.519", "O09.899", "O09.819", "O09.829", "O09.219", "O09.299", "O09.629", "O09.619", "0W8NXZZ", "10900ZC", "10903ZC", "10904ZC", "10907ZC", "10908ZC", "10D00Z0", "10D00Z1", "10D00Z2", "10D07Z3", "10D07Z4", "10D07Z5", "10D07Z6", "10D07Z7", "10D07Z8", "10S07ZZ", "10E0XZZ", "10D17Z9", "10D18Z9", "10A07ZZ", "10A08ZZ", "10A00ZZ", "10A03ZZ", "10A04ZZ", "10A07ZX", "10T20ZZ", "10T23ZZ" ,"10T24ZZ" ,"10D27ZZ" , "10D28ZZ","O60.23X9", "O60.23X5", "O60.23X4", "O60.23X3", "O60.23X2", "O60.23X1", "O60.23X0", "O60.22X9", "O60.22X5", "O60.22X4", "O60.22X3", "O60.22X2", "O60.22X1", "O60.22X0", "O69.9XX9", "O69.9XX5", "O69.9XX4", "O69.9XX3", "O69.9XX2", "O69.9XX1", "O69.9XX0", "O69.89X9", "O69.89X5", "O69.89X4", "O69.89X3", "O69.89X2", "O69.89X1", "O69.89X0", "O69.82X9", "O69.82X5", "O69.82X4", "O69.82X3", "O69.82X2", "O69.82X1", "O69.82X0", "O69.81X9", "O69.81X5", "O69.81X4", "O69.81X3", "O69.81X2", "O69.81X1", "O69.81X0", "O69.5XX9", "O69.5XX5", "O69.5XX4", "O69.5XX3", "O69.5XX2", "O69.5XX1", "O69.5XX0", "O69.4XX9", "O69.4XX5", "O69.4XX4", "O69.4XX3", "O69.4XX2", "O69.4XX1", "O69.4XX0", "O69.3XX9", "O69.3XX5", "O69.3XX4", "O69.3XX3", "O69.3XX2", "O69.3XX1", "O69.3XX0", "O69.2XX9", "O69.2XX5", "O69.2XX4", "O69.2XX3", "O69.2XX2", "O69.2XX1", "O69.2XX0", "O69.1XX9", "O69.1XX5", "O69.1XX4", "O69.1XX3", "O69.1XX2", "O69.1XX1", "O69.1XX0", "O69.0XX9", "O69.0XX5", "O69.0XX4", "O69.0XX3", "O69.0XX2", "O69.0XX", "O69.0XX0", "O36.4XX0", "O36.4XX1", "O36.4XX2", "O36.4XX3", "O36.4XX4", "O36.4XX5", "O36.4XX9", "O60.12X0", "O60.12X1", "O60.12X2", "O60.12X3", "O60.12X4", "O60.12X5", "O60.12X9", "O60.13X0", "O60.13X1", "O60.13X2", "O60.13X3", "O60.13X4", "O60.13X5", "O60.13X9", "O60.14X9", "O60.14X5", "O60.14X4", "O60.14X3", "O60.14X2", "O60.14X1", "O60.14X0")

*Intracranial infarct or hemorrhage ICD-10 codes:* "I60.", "I61.", "I62.", "I63.", "I65.", "I66.", "I67.", "I68.", "I69."

*Gastrointestinal hemorrhage ICD-10 codes:* "I60", "I61", "I62", "D62", "N02", "R31","R58", "S063","S064", "S065", "S066","I850", "K250", "K252", "K254","K256", "K260", "K262", "K264", "K266", "K270", "K272", "K274", "K276","K280", "K282", "K284", "K286", "K290", "K625", "K920", "K921", "K922","H113", "H356", "H431", "H450","H922", "J942", "K661", "M250", "N920", "N921", "N924", "N938", "N939","N950", "R040", "R041", "R042", "R048", "R049"

*Liver failure ICD-10 codes:* "K74.3", "K74.4", "K74.5", "K74.6", "K70.3", "K70.4", "K72.0", "K72.1", "K72.9"

*Surgical attending physician ICD-10 codes:* 4034, 4037, 4046, 4050

*Procedures for pulmonary embolism ICD-10 codes:* "02CP0ZZ","02CQ0ZZ","02CR0ZZ", "02CP3ZZ","02CQ3ZZ","02CR3ZZ","3E06317","6A750Z5","6A750Z6","6A750Z7","6A750ZZ","6A751Z5","6A751Z6","6A751Z7","6A751ZZ", "3E03317","3E04317"

*Other invasive procedures (lumbar puncture, paracentesis, abdominal wall drainage, thoracentesis, central line insertion) ICD-10 codes:* "009Y", "0W9G0Z", "0W9G3", "0W9F30", "0W993", "0W9B3", "05HN3", "05HM3", "06HM3", "06HN3", "05H53", "05H63"
