# Supplementary material for: Practice Patterns and Outcomes of Initial Anticoagulation Among Hospitalized Patients With Low- and Low-Intermediate-Risk Pulmonary Embolism
Source: CHEST Pulm. 2025 Feb 24;3(2):100151. doi: 10.1016/j.chpulm.2025.100151 (PMC12330347; doi:10.1016/j.chpulm.2025.100151)
Supplement: e-Online Data [file mmc2.docx]

**Practice Patterns and Outcomes of Initial Anticoagulation Among Hospitalized Patients With Low- and Low-Intermediate-Risk Pulmonary Embolism**

**Authors:**

Grace M. Ferri^1*^, Om A. Kothari^1*^, Sarika D. Gurnani^1^, Anica C. Law^2^, Nicholas A. Bosch^2^, Burton H. Shen^2^

**Supplemental Material:**

**e-Figure 1.** Sankey diagram illustrating mode of anticoagulation from admission to discharge.

Supplemental e-Figure 1 Legend: UFH = unfractionated heparin; Subcutaneous anticoagulation = low-molecular-weight heparin/fondaparinux.
